# Self-Reported Emotional States and Psychosocial Patterns in Patients With Active Venous Leg Ulcers (CEAP C6): A Descriptive Cross-Sectional Study

**DOI:** 10.7759/cureus.109652

**Published:** 2026-05-25

**Authors:** Fernando Vega-Rasgado, Lourdes A Vega-Rasgado, Fernando J Vega-Riveros, Alikvahan M Jiménez Corales, Fernando Contreras Cisneros

**Affiliations:** 1 Investigation, Instituto Mexicano de Flebología, México City, MEX; 2 Clinic, Instituto de Medicina Transpersonal, Oaxaca, MEX; 3 Neurochemistry Laboratory, Department of Biochemistry, National School of Biological Sciences, National Polytechnic Institute, México, MEX; 4 AI Laboratory, Instituto Mexicano de Flebología, México City, MEX; 5 Clinical Phlebology, Instituto Mexicano de Flebología, Oaxaca, MEX; 6 Investigation, Instituto Mexicano de Flebología, León Guanajuato, MEX

**Keywords:** allostatic load, biographical entrapment, chronic venous insufficiency, cvd, emotions, psychological stress, psychoneuroimmunology, quality of life, venous leg ulcer, wound healing

## Abstract

Introduction

Chronic venous disease (CVD), particularly in the class C6 ulcerative stage, imposes a profound multidimensional burden that extends beyond hemodynamic dysfunction. While the biological basis of venous leg ulcers (VLUs) is well-characterized, the integration of psychosocial states within a psychoneuroimmunology (PNI) framework remains exploratory. This study aimed to characterize self-reported emotional trajectories and exploratory psychosocial indicators in patients with active VLUs.

Methods

A descriptive cross-sectional study was conducted in Mexico with 30 patients presenting active VLUs, categorized according to the Clinical, Etiological, Anatomical, and Pathophysiological (CEAP) classification system as class C6. Participants were assessed using a structured survey to map 15 emotional states and specific psychosocial constructs across three clinical milestones: a recalled pre-morbidity baseline, the current active ulcer stage, and a hypothetical healing projection. Data were analyzed to identify descriptive patterns of psychological distress and psychosocial well-being.

Results

A marked descriptive shift in emotional distribution was observed across reported milestones. The transition from baseline to the active ulcer stage was characterized by a prominent surge in aggregated psychological distress from 8.8% to 55.5% (+46.7 percentage points). During active ulceration, the most frequent states were shame (90.0%), vulnerability (70.0%), sadness (66.7%), fear (60.0%), and anxiety (56.7%). High frequencies were also observed for exploratory psychosocial indicators, specifically the high-responsibility profile (86.7%, *n* = 26) and perceived entrapment (76.7%, *n *= 23). In the hypothetical healing scenario, psychosocial well-being increased to 78.3% (+69.1 percentage points).

Conclusions

Patients with active class C6 venous ulcers demonstrate a high prevalence of emotional burden and specific psychosocial profiles related to perceived obligation and entrapment. Their most prevalent emotional states include shame, vulnerability, sadness, fear, and anxiety. These findings suggest that incorporating psychological assessment and support could be considered a potentially relevant component of comprehensive care for these patients, alongside traditional hemodynamic management. However, as these are hypothesis-generating observations, longitudinal research is required to determine the clinical impact of such interventions on wound healing trajectories and therapeutic engagement.

## Introduction

Chronic venous disease (CVD) is highly prevalent worldwide, with population-based studies and reviews estimating prevalence figures between 10% and 60%, depending on case definition and age group. Venous leg ulcers (VLUs) represent 75-80% of all lower limb ulcers [1,2]. While its multifactorial etiology (including genetic predisposition, age, gender, and environmental factors) is well established, VLU constitutes both a clinical challenge and a multidimensional burden for the patient [3].

From a pathophysiological standpoint, CVD is characterized by valvular incompetence and venous hypertension, leading to progressive skin changes and, in advanced stages, chronic ulceration that may be difficult to heal [1]. However, hemodynamic dysfunction alone may not fully account for the patient experience associated with chronic wounds [3]. Within a biopsychosocial framework, emotional burden, illness perception, and psychosocial context are increasingly recognized as relevant dimensions of how chronic disease is experienced and managed [3].

The impact of CVD, particularly in the ulcerative stage, extends beyond physical impairment. Chronicity, persistent pain, exudate, and malodor have been reported to impose a substantial emotional burden [4]. Previous research has documented psychiatric comorbidity and deterioration in quality of life (QoL) among patients with VLUs [5]. Furthermore, elevated levels of anxiety and depression have also been described, together with impaired self-image and reduced social participation [4,6]. Different psychometric instruments, including the Venous Leg Ulcer Quality of Life Questionnaire (VLU-QoL), have been used to quantify this burden [7]. Notably, depressive symptoms have also been reported in earlier stages of CVD [8].

Psychoneuroimmunology (PNI) provides one possible conceptual framework for discussing the relationship between emotional factors and chronic disease, as it examines interactions among the nervous, endocrine, and immune systems [9]. The literature has proposed that chronic stress and persistent negative emotional states may influence disease-related processes through dysregulation of the hypothalamic-pituitary-adrenal (HPA) axis and related inflammatory pathways [10]. Illness perception has likewise been associated with health outcomes in different chronic conditions [11,12], and beliefs of uncontrollability or fatalism may influence distress and engagement with treatment [13]. In the present study, these frameworks are used only as a conceptual background, as no biological mediators were measured.

The objective of this preliminary cross-sectional descriptive study was to describe the frequency of certain self-reported emotional states and exploratory psychosocial aspects in patients with active venous ulcers, categorized as class C6 according to the Clinical, Etiological, Anatomical, and Pathophysiological (CEAP) classification system. Furthermore, the study characterized their distribution across a recalled baseline period, the current ulcer stage, and a hypothetical healing scenario. The aim was not to demonstrate associations or causal mechanisms, but rather to provide descriptive observations that could inform future hypothesis-driven research involving emotions.

## Materials and methods

Study design and population

This preliminary descriptive cross-sectional study was conducted across several medical centers in México, focusing on a total cohort of 30 patients with active venous ulceration (CEAP C6). Participants were enrolled through a convenience, non-probabilistic sampling strategy. The study was designed to provide a descriptive characterization of self-reported emotional states and exploratory psychosocial aspects in the context of advanced venous disease.

Eligible participants were adults (≥18 years) with clinically confirmed CEAP C6 ulcers, cognitive competence, and sufficient communicative ability to respond to the clinician-administered survey. To minimize confounding variables, exclusion criteria included significant cognitive impairment, active psychotic or severe personality disorders, critical limb ischemia (ankle-brachial index < 0.5), and terminal illness.

Ethics statement

The study protocol was reviewed and approved by the Institutional Review Board of the Instituto Mexicano de Flebología, Ethics and Research Committee (approval no. IMF-CEI-01/2024). A waiver of written informed consent was approved by the committee in order to preserve anonymity, and participation proceeded through a documented verbal informed consent process during clinical consultations, in accordance with the Declaration of Helsinki.

Measurement instrument

Psychosocial factors were assessed using the “Venous Disease and Emotions Survey,” an exploratory clinician-administered instrument developed by the authors to characterize the multidimensional psychosocial burden associated with active venous ulcers (see Appendix). The survey was designed as a hypothesis-generating clinical tool rather than a validated psychometric scale intended for confirmatory measurement.

Two exploratory psychosocial aspects of primary interest were operationalized as clinical indicators: “perceived entrapment,” defined as reported chronic stress associated with a perceived lack of autonomy or personal agency, and the “high-responsibility profile,” defined as the perception of a sustained and indispensable systemic or family support role. Additional exploratory indicators included psychosocial stagnation and symptom-linked identity. Table 1 summarizes the operational definitions used during clinical assessment.

**Table 1:** Operational definitions of exploratory psychosocial indicators assessed in the “Venous Disease and Emotions Survey” These aspects were operationalized into specific clinical indicators to facilitate systematic recording during clinical consultations. The "Venous Disease and Emotions Survey" is an exploratory, clinician-administered instrument designed for hypothesis generation; it is not a validated psychometric scale for confirmatory measurement.

Indicator label	Clinical operational definition	Sample survey item
High-responsibility profile	Perception of a sustained and indispensable systemic or family support role.	“Do you feel that if you stop making an effort, your family or work system would collapse?”
Perceived entrapment	Reported chronic stress associated with a perceived lack of autonomy or personal agency.	“Do you feel your current situation is like a dead end from which you cannot escape?”
Psychosocial stagnation	Perception of a halt in personal development or biographical progression.	“Do you feel that your life is on pause or that you have not made progress in years?”
Symptom-linked identity	Perception of the disease as a primary defining factor of the patient’s current social role.	“Do you feel that the ulcer has become the only way others recognize your needs?”

Emotional domain and reported milestones

The instrument assessed individual self-reported emotional states across three reported milestones: a recalled pre-morbidity baseline, the current active ulcer stage, and a hypothetical healing scenario. These milestones were used to describe the distribution of self-reported emotional states across recalled, current, and projected clinical contexts. The recalled baseline was retrospective, and the healing scenario represented an imagined recovery state rather than an observed longitudinal outcome.

For descriptive visualization only, 15 individual emotional states were grouped into two predefined domains: psychological distress, comprising 11 negative variables (sadness, fear, anxiety, anger, guilt, vulnerability, shame, hopelessness, depression, nostalgia, and boredom), and psychosocial well-being, comprising four positive indicators (joy, happiness, peace, and excitement). These descriptive domains were used to summarize broad emotional patterns and should not be interpreted as validated composite scores.

Statistical analysis

Data management and analysis were performed using Microsoft Excel (Version 2024, Microsoft Corp., Redmond, WA). Given the exploratory nature of this preliminary study and the sample size (*n* = 30), the statistical approach was descriptive only. Categorical variables were summarized as absolute frequencies and percentages, and continuous variables were summarized as mean ± standard deviation (SD). Absolute differences across reported milestones were expressed in percentage points (Δ pp). Consolidated percentages for the descriptive emotional domains were calculated as the average proportion of endorsed items within each predefined domain and were used exclusively for descriptive visualization. No inferential association testing was performed.

## Results

Sample characteristics and comorbidities

The study included 30 patients with active venous ulcers (CEAP C6). In this sample, 60.0% of patients were women, the mean age was 59.33 ± 15.4 years, and the mean duration of active ulceration was 14.5 months (range, 1-120 months). Overweight was present in 53.3% of cases (*n* = 16), controlled hypertension in 36.7% (*n* = 11), and controlled diabetes mellitus in 23.3% (*n* = 7). Detailed sociodemographic and comorbidity characteristics are summarized in Table 2.

**Table 2:** Sociodemographic and comorbidity characteristics of the study sample (n = 30). Categorical variables are expressed as *n* (%). Continuous variables are expressed as mean ± SD.

Characteristic	Study sample (n = 30), n (%)
Male sex	12 (40)
Female sex	18 (60)
Age (years)	59.33 ± 15.4
Comorbidities	
Overweight	16 (53.3)
Hypertension (controlled)	11 (36.7)
Diabetes mellitus (controlled)	7 (23.3)
Ulcer duration (months)	14.5 (range: 1-120)

Distribution of self-reported emotional states across reported milestones

To facilitate descriptive visualization, the 15 individual emotional states assessed in this sample were grouped into two descriptive domains: *Psychological Distress* and *Psychosocial Well-being*. The item-level distribution of these 15 emotional states across the three reported milestones is shown in Table 3.

**Table 3:** Distribution of individual self-reported emotional states across reported milestones in the study sample (n = 30) The recalled baseline represents a retrospective pre-morbidity state. The active ulcer stage reflects the patient’s current clinical status, and the hypothetical healing scenario represents a projected state rather than an observed outcome.

Emotional state	Recalled baseline (retrospective), n (%)	Active ulcer stage, n (%)	Hypothetical healing scenario, n (%)
Sadness	3 (10.0)	20 (66.7)	2 (6.7)
Fear	4 (13.3)	18 (60.0)	3 (10.0)
Anxiety	5 (16.7)	17 (56.7)	5 (16.7)
Anger	2 (6.7)	14 (46.7)	1 (3.3)
Guilt	1 (3.3)	12 (40.0)	0 (0.0)
Vulnerability	2 (6.7)	21 (70.0)	2 (6.7)
Shame	1 (3.3)	27 (90.0)	4 (13.3)
Hopelessness	0 (0.0)	15 (50.0)	1 (3.3)
Depression	2 (6.7)	13 (43.3)	1 (3.3)
Nostalgia	8 (26.7)	10 (33.3)	2 (6.7)
Boredom	1 (3.3)	16 (53.3)	3 (10.0)
Joy	11 (36.7)	4 (13.3)	22 (73.3)
Happiness	10 (33.3)	3 (10.0)	23 (76.7)
Peace	9 (30.0)	2 (6.7)	25 (83.3)
Excitement	8 (26.7)	2 (6.7)	24 (80.0)

**Figure 1:**
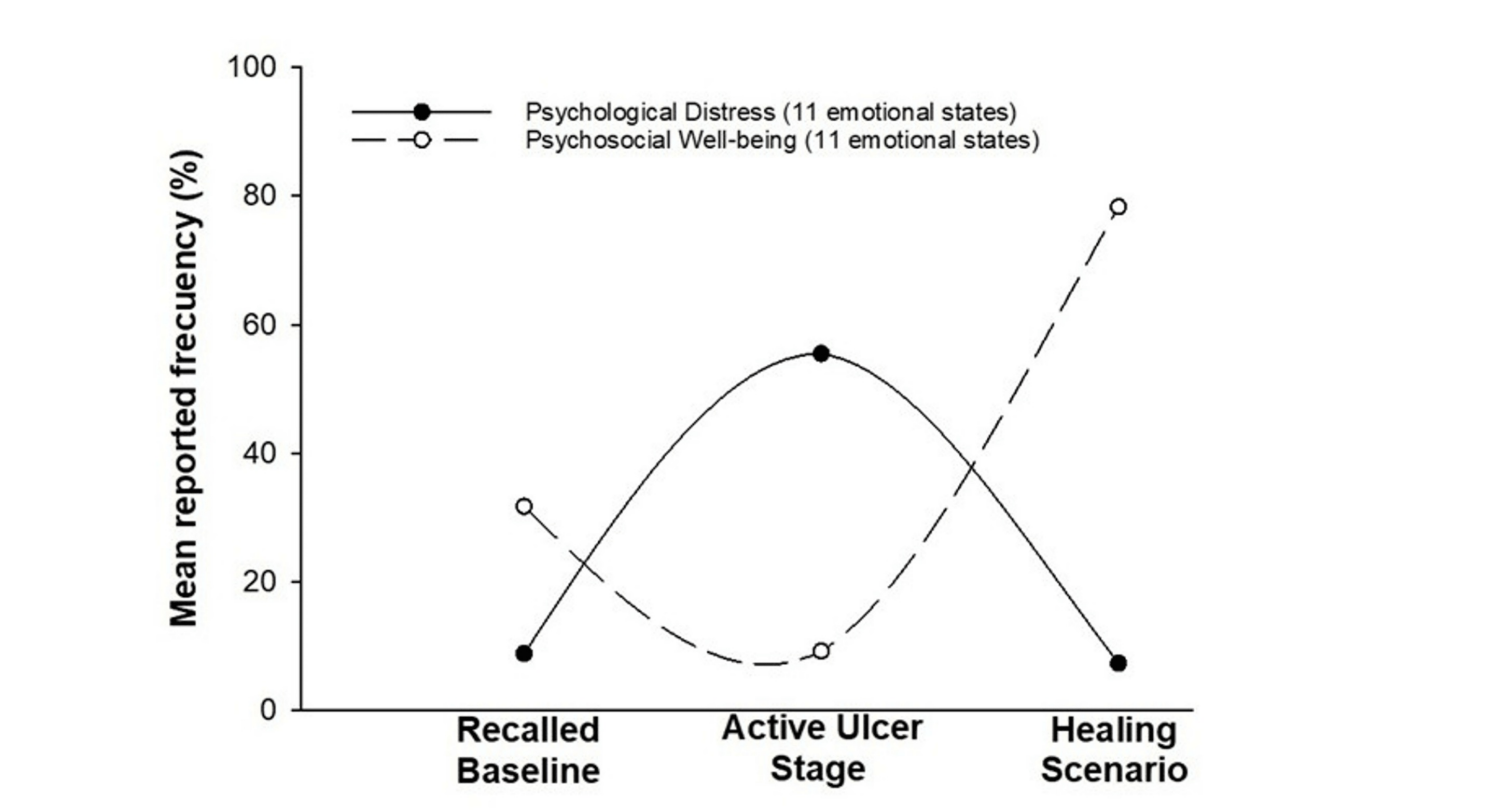
Patient-reported emotional trajectories across perceived clinical milestones in active venous ulceration (CEAP C6) The figure illustrates the frequencies of consolidated emotional constructs across recalled pre-morbidity baseline, current active ulcer stage, and a hypothetical healing scenario. The graphic provides a descriptive visualization of the reversal in emotional polarity associated with clinical status.

Across the reported milestones, a descriptive shift in emotional distribution was observed. The predefined domain labelled *Psychological Distress* increased from 8.8% at the recalled baseline to 55.5% during the active ulcer stage, corresponding to an absolute difference of +46.7 percentage points. By contrast, the predefined domain labelled *Psychosocial Well-being* increased from 9.2% during the active ulcer stage to 78.3% in the hypothetical healing scenario, corresponding to an absolute difference of +69.1 percentage points. At the active ulcer stage, the most frequently reported negative emotional states were shame (90.0%), vulnerability (70.0%), sadness (66.7%), fear (60.0%), and anxiety (56.7%). These descriptive patterns are summarized in Table 4 and illustrated in Figure 1.

**Table 4:** Summary of the consolidated emotional constructs and magnitude of change across clinical milestones (n = 30). Psychological distress represents the mean frequency of 11 negative emotional dimensions, and psychosocial well-being represents the mean frequency of four positive emotional dimensions. Δ pp: Difference expressed in percentage points. These values were used exclusively for descriptive visualization and do not represent validated composite scores.

Emotional domain	Recalled baseline	Active ulcer stage	Hypothetical healing scenario	Δ pp (active vs. baseline)	Δ pp (projection vs. active)
Psychological distress	8.8%	55.5%	7.3%	+46.7	-48.2
Psychosocial well-being	31.7%	9.2%	78.3%	-22.5	+69.1

Exploratory psychosocial aspects

**Figure 2:**
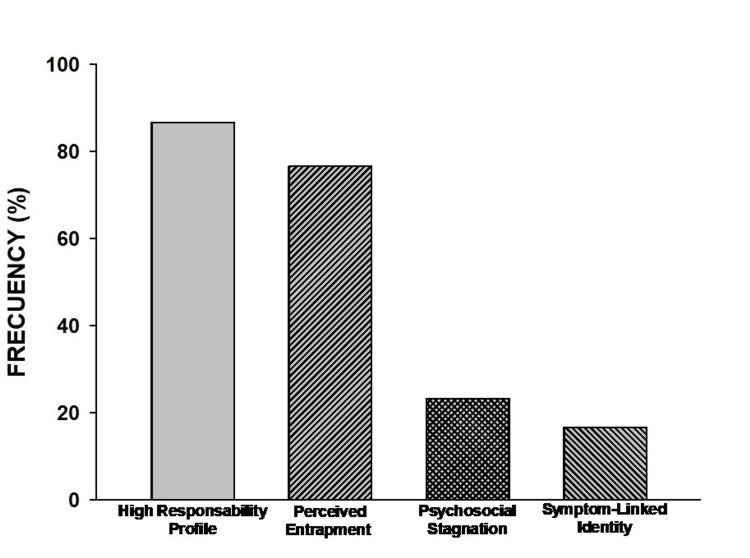
Most prevalent self-reported negative emotional states in patients during the active ulcer stage (CEAP C6). Frequencies are based on a sample of n = 30 patients. These indicators are exploratory and derived from the clinician-administered “Venous Disease and Emotions Survey.” They are intended for hypothesis generation and do not constitute a validated psychometric scale for confirmatory measurement.

High reported frequencies were observed for the high-responsibility profile (86.7%, *n* = 26) and perceived entrapment (76.7%, *n* = 23), the two most prominent exploratory psychosocial indicators in this sample. Other exploratory indicators, such as psychosocial stagnation (23.3%, *n* = 7) and symptom-linked identity (16.7%, *n* = 5), were less frequent (Figure 2).

**Figure 3:**
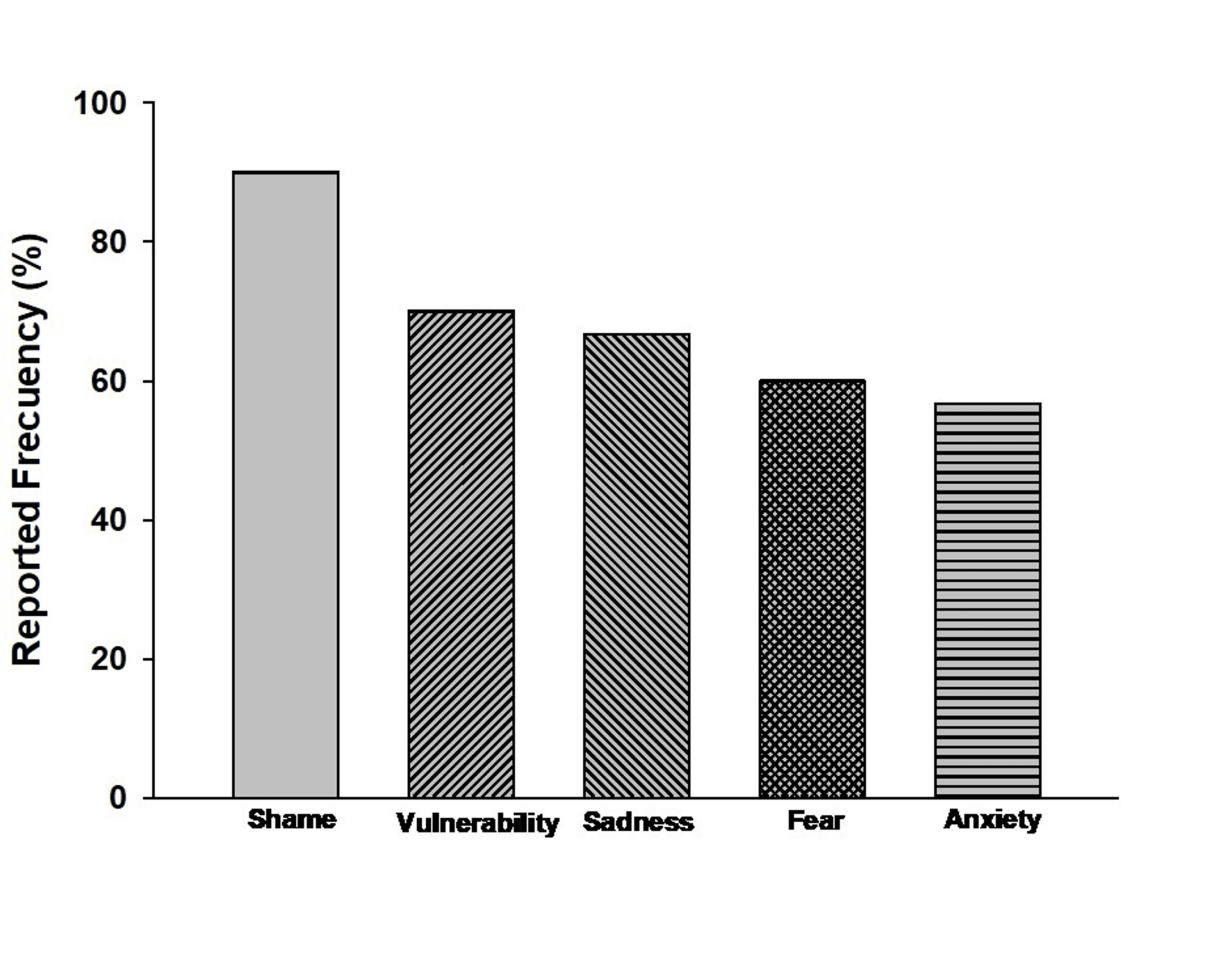
Prevalence of self-reported negative emotional states during the active ulcer stage (CEAP C6). Frequencies are based on a sample of *n* = 30 patients. The figure displays the four negative emotional states with the highest reported prevalence during active ulceration. These data are exploratory, collected via the clinician-administered “Venous Disease and Emotions Survey,” and are intended for descriptive comparison and hypothesis generation.

During the active ulcer stage, shame (90.0%), vulnerability (70.0%), sadness (66.7%), fear (60.0%), and anxiety (56.7%) were the most prevalent self-reported negative emotional states. These results are presented in Figure 3 alongside selected exploratory psychosocial indicators to facilitate descriptive comparison.

## Discussion

This preliminary descriptive cross-sectional analysis highlights a substantial self-reported emotional burden in 30 patients with active venous ulcers (CEAP C6). The main findings were the high reported frequencies of shame, vulnerability, sadness, fear, and anxiety during the active ulcer stage, together with the frequent presence of two exploratory psychosocial indicators, namely, the high-responsibility profile and perceived entrapment. These observations are presented as descriptive findings intended for hypothesis generation rather than as evidence of direct causal or mechanistic relationships. Table 5 summarizes relevant literature regarding the relationship between psychological states and wound healing.

**Table 5:** Key literature investigating the relationship between psychological morbidity and clinical outcomes in chronic wounds. Summary of selected literature investigating correlations between psychological morbidity, emotional distress, and clinical outcomes in chronic wounds. This table is provided as explanatory background for the Discussion and does not represent direct measurements obtained in the present study.

Psychological dimension	Physiopathological impact / mechanism	Reference (authors, year)
Anxiety and depression	Level I evidence: significant correlation between distress and delayed healing outcomes	O’Donovan et al. (2024) [14]
Chronic stress	Sustained HPA axis activation; elevated cortisol levels; proliferative phase delay	Blackburn-Munro (2001) [10]
Social distress	Pro-inflammatory cytokine modulation (IL-6, CRP), prolonging chronic inflammation	Kiecolt-Glaser et al. (2002) [9]
Illness perception	Low self-efficacy and maladaptive behaviors impacting recovery expectations	Walburn et al. (2009) [15]
Wound-related stress	Dysregulation of MMP-9 (matrix metalloproteinase); extracellular matrix degradation	Woo et al. (2024) [16]
Neuroendocrine axis	β2-adrenergic receptor-mediated inhibition of keratinocyte migration	Sivamani et al. (2009) [17]
Hypervigilance	Focus on odor and pain; neurobiological alert state causing severe QoL reduction	Weir & Davies (2023) [18]
Social rejection	Activation of the d-ACC; neurobiological overlap between social and physical pain	Eisenberger (2012) [19]

The observed emotional burden is broadly consistent with prior literature showing that chronic wounds and venous ulcers are associated with impaired quality of life, psychiatric comorbidity, anxiety, depressive symptoms, impaired self-image, and reduced social participation [4,5,6]. In the present sample, shame and vulnerability were especially prominent during the active ulcer stage, while the High-Responsibility Profile and Perceived Entrapment offered descriptive anchors for how patients narrated burden, obligation, and constraint in the context of active ulceration. While previous literature on venous leg ulcers has focused predominantly on characterizing anxiety and depression, the present study expands the emotional scope to include a broader spectrum of states [15]. Notably, our findings indicate that emotions such as shame and vulnerability reached higher reported frequencies than conventional anxiety and depression, highlighting a more complex psychosocial burden in CEAP C6 patients than previously recognized [19].

The exploratory psychosocial indicators in this study should be interpreted within the limits of their operational definitions rather than as validated psychosocial phenotypes. Their value in the present report is descriptive: they help characterize recurring patterns in how patients narrated burden, obligation, and constraint while living with active venous ulceration. By contrast, shame and vulnerability should be interpreted as prominent negative self-reported emotional states rather than as exploratory psychosocial indicators. The marked increase in descriptive Psychological Distress and the frequency of Perceived Entrapment are compatible with the idea of venous ulceration as a form of biographical disruption, and similar patterns have been associated in prior literature with delayed tissue repair and a pro-inflammatory clinical background [14,20,21].

PNI remains relevant to the interpretation of these findings as a literature-based explanatory framework, even though the present study did not directly measure biological mediators. In the original conceptual background of this manuscript, PNI was used to contextualize potential interactions among the nervous, endocrine, and immune systems, including hypothalamic-pituitary-adrenal axis dysregulation, chronic stress responses, inflammatory modulation, and illness perception [9,10,11,12,13]. Within that framework, the prominence of shame (90.0%), vulnerability (70.0%), sadness (66.7%), fear (60.0%), anxiety (56.7%), the high-responsibility profile (86.7%), and perceived entrapment (76.7%) may be interpreted as clinically meaningful because they are compatible with stress-related and illness-perception mechanisms discussed in the chronic wound literature [14,15].

The present descriptive findings may also be contextualized through the concept of allostatic load [22]. The persistent nature of the ulcer is interpreted as a chronic stressor under the framework of the general adaptation syndrome, where exhaustion of homeostatic regulation translates into cumulative allostatic burden [22,23]. In this sample, the combination of high frequencies of shame, vulnerability, and entrapment (together with the surge in psychological distress) justifies discussing these constructs as possible psychosocial correlates of cumulative stress. Furthermore, the frequent High-Responsibility Profile may be clinically relevant, as it could reflect a persistent perception of obligation and vigilance in patients who perceive themselves as indispensable within their systems [22, 23].

From the perspective of prior PNI and wound-healing research, several biological pathways have been proposed to explain how psychological distress might be clinically relevant to ulcer chronicity. These include hypothalamic-pituitary-adrenal (HPA) axis activation and elevated cortisol levels [10], dysregulation of matrix metalloproteinases [16], and impaired keratinocyte migration through β2-adrenergic signaling [4,9,17,24,25]. Furthermore, research suggests that reduced collagen-related repair signaling [26] may lead to a significant decrease in extracellular matrix synthesis. Additional proposed pathways include the persistence of pro-inflammatory macrophage activity [4,27] and the functional overlap between social distress and pain-related neural circuits, such as the dorsal anterior cingulate cortex (dACC) [28,29].

However, this explanatory framework should not be confused with direct inference from the current study. The present analysis did not assess cortisol, cytokines, macrophage polarization, matrix metalloproteinases, collagen synthesis, keratinocyte migration, or neuroimaging correlates; therefore, it cannot demonstrate that the reported emotional states or exploratory psychosocial indicators caused delayed healing or mediated biological dysregulation in this sample. Rather, the PNI literature helps situate the descriptive findings within a broader biopsychosocial context and provides biologically plausible hypotheses for future longitudinal or translational research.

An additional observation from this study was the marked increase in aggregated positive emotional states under the hypothetical healing scenario. Because this scenario was projected rather than observed, it should be interpreted cautiously. Nevertheless, the magnitude of this shift suggests that patients anticipate a substantial emotional recovery following wound resolution. This projected reversibility aligns with hypotheses suggesting that hope-related states may interact with neurobiological stress regulation and tissue-repair processes [30]. In the present report, however, this projection is treated as a descriptive reflection of patient expectations rather than evidence of a demonstrated neurobiological reversal.

From a clinical perspective, the present findings support the value of recognizing emotional burden and psychosocial context during the assessment of patients with active venous ulcers. They do not establish validated psychosocial phenotypes, nor do they demonstrate associations with healing outcomes. Rather, they suggest that structured attention to self-reported distress, perceived burden, illness-related life impact, and the broader biopsychosocial context may enrich the clinical understanding of advanced venous disease and help inform future multidisciplinary research [3].

This study has important limitations. First, the sample was small (*n* = 30), which limits precision and generalizability. Second, participants were enrolled using convenience sampling, which increases the risk of selection bias. Third, the survey instrument was exploratory and clinician-administered, and it was not psychometrically validated for confirmatory measurement. Fourth, the exploratory psychosocial indicators were based on operational clinical definitions and should not be interpreted as validated constructs. Fifth, the recalled baseline was retrospective and therefore subject to recall bias, while the healing scenario was hypothetical rather than an observed longitudinal outcome. Sixth, the aggregated emotional domains were descriptive summaries only and should not be interpreted as validated composite scores. Finally, no inferential association testing or biological mediator assessment was performed, so any PNI-related interpretation remains literature-based and hypothesis-generating.

## Conclusions

In this preliminary descriptive cross-sectional sample of 30 patients with active venous ulcers (CEAP C6), the most prevalent emotional states were shame, vulnerability, sadness, fear, and anxiety. High-responsibility profile and perceived entrapment emerged as frequent psychosocial indicators. These findings suggest the value of incorporating psychological assessment and support alongside traditional hemodynamic management for patients with VLUs. However, the extent to which such interventions might influence clinical outcomes or wound healing remains an open question to be addressed through future interventional research. Given the exploratory nature of this study, these findings are intended to generate hypotheses rather than to establish definitive clinical protocols.

## Appendices

**Table 6:** Venous disease and emotions survey instrument Note: This survey was originally developed in Spanish and translated into English for the purpose of this publication, ensuring semantic equivalence. This instrument is designed as an analytical and exploratory framework rather than a definitive psychometric scale; its primary goal is to identify complex psychosocial constructs (such as "perceived entrapment" and the "high-responsibility profile") that are frequently overlooked in standard clinical reports. As an evolving tool within psychoneuroimmunology, it facilitates the inferential mapping of emotional trajectories and remains subject to further iterative refinement and broader psychometric validation.

Dimension / category	Survey item (question)	Response format
Sociodemographic Profile	Gender, age, geographic location, area of residence (rural, semirural, urban), living situation (couple, family, single)	Multiple choice / numeric
Clinical & Lifestyle Background	Perceived weight (normal, overweight, etc.), occupation and physical activity (sitting, standing, walking), time since disease onset	Multiple choice / categorical
Disease Severity	Clinical, Etiology, Anatomy, and Pathophysiology (CEAP) classification (C1 to C6); associated comorbidities (hypertension, diabetes, colitis, heart diseases, gastritis, thyroid disease, depression, etc.)	Categorical / checklist
Social & Self-Stigma (Varicose Veins)	Feelings of embarrassment (visibility to others), social restriction, impact on self-esteem, indifference to appearance	Checklist (multiple selection)
Social & Self-Stigma (Active Ulcer)	Sadness regarding visibility, embarrassment of bandages, social restriction, impact on self-esteem	Checklist (multiple selection)
Emotional Spectrum (Current State)	"What emotions does suffering from ulcers or varicose veins produce in you?" (e.g., joy, sadness, fear, anxiety, vulnerability, etc.).	Checklist of 17 emotions + "Other"
Healing Projection & Resilience	"What would you do if you didn't have an ulcer/varicose veins?", "If your condition has healed, how do you feel?".	Open-ended / emotional checklist
Qualitative/Narrative Inquiry	"Would you like to share a problem that causes you to not be calm or at peace? Describe it."	Open-ended (narrative)
